# Self-reported prevalence of periodontal disease among the Spanish population and immigrants: 2006, 2011/12 and 2017: a population-based study

**DOI:** 10.1186/s12903-021-01579-z

**Published:** 2021-04-28

**Authors:** Diego Gómez-Costa, Jesús San-Roman-Montero, Rosa Rojo, Ángel Gil, Rafael Gómez de Diego, Antonio F. López-Sánchez

**Affiliations:** 1 Doctoral Program in Health Sciences, Faculty of Health Sciences, Rey Juan Carlos University, Avenida de Atenas s/n, Alcorcón, 28922 Madrid, Spain; 2 Department of Medicine Specialties and Public Health, Faculty of Health Sciences, Rey Juan Carlos University, Avenida de Atenas s/n, Alcorcón, 28922 Madrid, Spain; 3 Faculty of Dentistry, Alfonso X El Sabio University, Villanueva de la Cañada, 28691 Madrid, Spain; 4 Department of Nursing and Estomatology, Faculty of Health Sciences, Rey Juan Carlos University, Avenida de Atenas s/n, Alcorcón, 28922 Madrid, Spain

**Keywords:** Periodontal disease, Survey, Immigrants, Population study, Self-reported

## Abstract

**Background:**

Periodontal disease is one of the most common pathologies in the population. Self-reporting has been used as a diagnostic tool in large populations, among other reasons, to detect the needs of potentially vulnerable groups. This study evaluated the prevalence of periodontal disease in people of Spanish nationality and immigrants in Spain.

**Methods:**

This population-based, cross-sectional study was carried out using data obtained from National Health Interview Surveys (NHSs) carried out in 2006, 2011/2012 and 2017 in Spain. Subjects aged 16 years and older were included in the NHS-2006 and aged 15 years and older were included in the other NHSs. The following variables were self-reported by the participants: gum bleeding, tooth mobility, tooth extraction and missing teeth. Chi-square homogeneity tests were performed to assess the main associations between the independent variable (nationality) and the dependent variables (bleeding gums, tooth mobility, tooth extraction and missing teeth). Multinomial logistic regression models were constructed to evaluate the influences of the variables age and sex and their interactions on the main associations.

**Results:**

A total of 115,123 participants were included in the NHS-2006 (n = 37,327, 11.38% immigrants), NHS-2011/12 (n = 38,727, 14.39% immigrants) and NHS-2017 (n = 39,069, 13.71% immigrants). The variables directly related to periodontal disease were gum bleeding and tooth mobility. These were significantly associated with nationality in the NHS-2006 and NHS-2017 cohorts. In the NHS-2011/12 cohort, only tooth mobility was associated with nationality. After adjustments for sex, age, and their interactions, immigrant status was associated with increased odds of bleeding in only the NHS-2006 cohort (RR = 1.65, 95% CI 1.38–1.99, *p* = 0.000).

**Conclusion:**

Immigrants in Spain have a lower probability of developing signs associated with periodontal disease than the Spanish population. Among the immigrant cohort, females and those in adult age groups had lower prevalence rates than their counterparts.

## Background

The most investigated periodontal diseases [1] have been gingivitis, whose prevalence is almost universal, and periodontitis, whose distribution depends on the case definition, with partial mouth or index recording. These categories have tended to underestimate the prevalence of periodontitis and may affect approximately 11.2% of the world population, becoming a global health problem. [2]. The prevalence fluctuates among countries and regions [3, 4]. Moreover, certain local and genetic factors are related to its development, but there are other determinants, such as demographic and lifestyle factors, that are considered potential risk factors [5].

An individual’s perception of their health status has been advocated as a useful diagnostic tool in investigations of different health and disease states, especially when the objective is to assess a large study population. With the use of this subjective measure, an individual's health status and functional capacity can be determined [6]. Self-reported tools have recently been used in Spain [7, 8] and in neighboring countries such as Portugal [9, 10] to assess the prevalence of periodontitis in the general population. However, perceptions can vary depending on social groups [11]. In societies characterized by continuous mobility, the reception of immigrants in different countries may differ, and immigrants represent a group susceptible to social vulnerability in relation to possible health needs [12].

Some studies have evaluated oral health indicators, observing positive effects with regard to the care of immigrants [13]. However, several studies have reported a positive relationship between immigrant status and poor oral health [14]. For this reason, it is important to explore the situation in immigrant cohorts in different countries.

In Spain, oral care in autonomous communities follows public and mixed-care models. In the public health system, only children receive preventive and restorative treatments involving permanent dentition free of charge. Furthermore, these measures are provided inconsistently among different autonomous communities. The health administration does not take responsibility for care and prevention in those aged less than 6 years, young adults, elderly individuals, people with physical or behavioral limitations who cannot receive outpatient care, medically compromised people and immigrants [15–17].

Various studies have been carried out with the aim of comparing oral health conditions in immigrant groups with those in the Spanish population, but most of these studies have been carried out in only young people and/or children [18, 19].

The objective of this study was to compare the prevalence of periodontal disease between people of Spanish nationality and immigrants in Spain.

## Methods

A population-based, cross-sectional study was conducted following the Strengthening the Reporting of Observational studies in Epidemiology (STROBE) [20] guidelines.

### Setting, participants and study size

Data were obtained from three Spanish National Health Surveys (NHSs) conducted in 2006, 2011/12 and 2017.

The information collection periods were from June 2006 to June 2007 (NHS-2006), from July 2011 to June 2012 (NHS-2011/12) and from October 2016 to October 2017 (NHS-2017). Household questionnaires were administered throughout the national territory to those over 16 years of age in the NHS-2006 and those over 15 years of age in the other surveys (NHS-2011/12 and NHS-2017).

Sample selection was carried out by stratified random sampling of autonomous communities, selecting one part of the sample uniformly and the other part of the sample proportionally to the size of the community. The sample size was calculated by the National Institute of Statistics. The surveys were directed to the group of people residing in main family dwellings. When a dwelling consisted of two or more households, the study was extended to all of them, but independently for each household. The initial contact with the selected households was made by sending a letter from the Ministry of Health, Consumption and Social Welfare MSCBS. The information collection method was carried out employing a personal computer-assisted interview that could be supplemented, when necessary and in exceptional cases, through a telephone interview. The methodological details are accessible for public use [21, 22].

### Variables

The only sociodemographic variables collected in the three surveys were sex, age, and nationality (Spanish or foreign). The variables related to periodontal disease were self-reported variables; participants were asked about the health status of their teeth and molars with regard to the following: (1) gum bleeding (“gums bleed when brushing or *spontaneously*”), (2) dental mobility (“teeth and/or molars move"), (3) tooth exodontia ("teeth and/or molars have been extracted") and (4) dental absence ("missing teeth and/or molars that have not been replaced by prostheses").

All the variables collected were categorical variables. The independent variable in this study was nationality, and the dependent variables were those related to periodontal disease. Age and sex were considered modifying (or interaction) variables.

### Ethical aspects

Patient information was anonymized and deidentified prior to analysis. The local ethics committee (the Rey Juan Carlos University Research Ethics Committee) ruled that no formal ethical approval was required for this study.

### Statistical methods

Descriptive statistics include the calculations of the frequencies and the percentages of each of the variables. The Shapiro–Wilk test was used to assess the normality of the data. An analysis of missing data was carried out, considering that variables with values lower than 10% could be decisive for the statistical analysis, in which case they would be excluded from the study. Missing values, recorded under the category “not recorded,” were only used for descriptive statistics. Chi-square tests of homogeneity were performed to evaluate the main associations between the independent variable (nationality) and dependent variables (bleeding gums, tooth mobility, tooth extraction, or tooth absence). Multinomial logistic regression models were constructed to evaluate the influence of age, sex and their interaction on the main association. The interactions of nationality and sex as well as nationality and age were evaluated with the likelihood ratio test. In the case of significant results, the interactions were included in the models. STATA® 14 (StataCorp, College Station, TX, USA) was used for all tests. The values were considered statistically significant at *p* < 0.05.

## Results

**Fig. 1:**
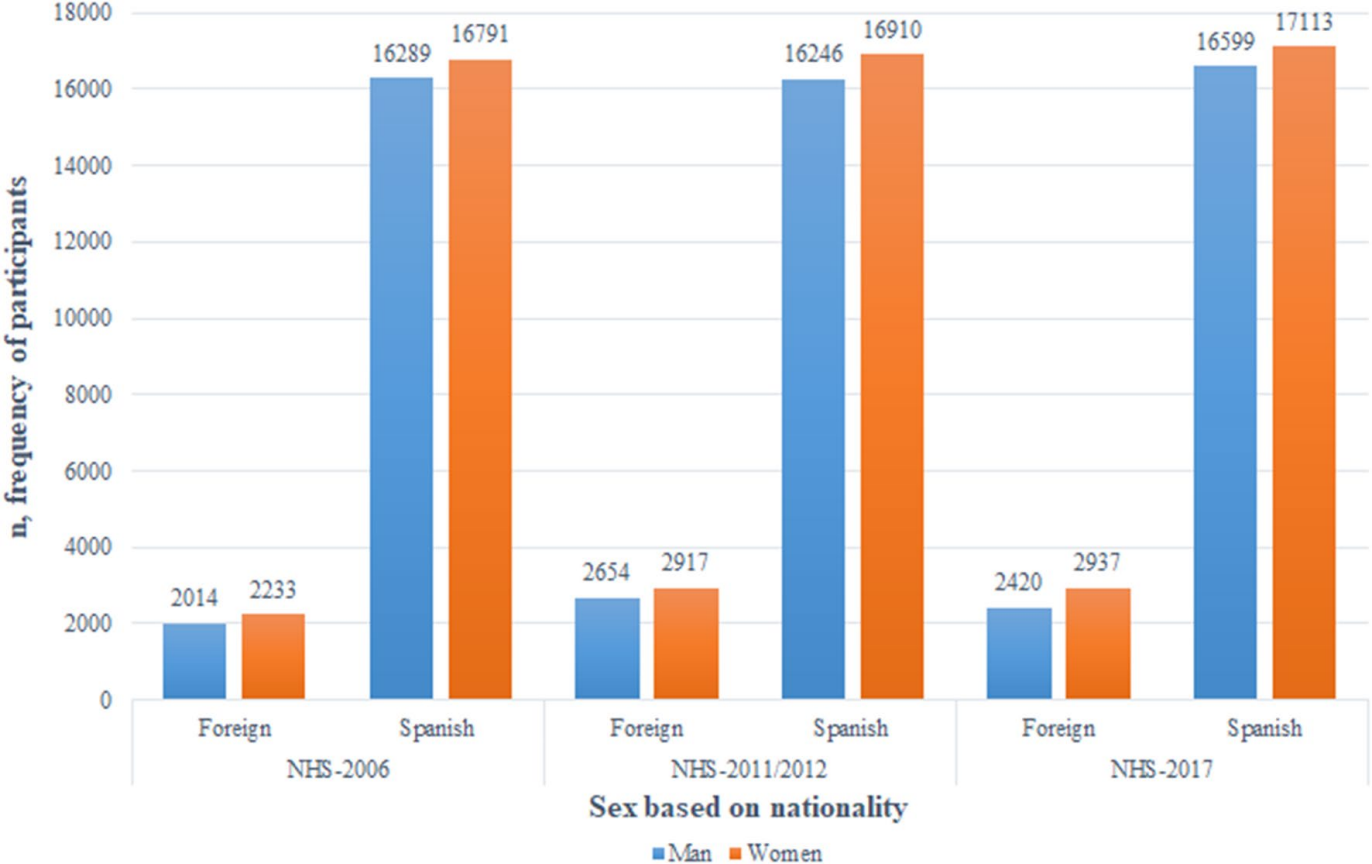
Distributions of the participants according to sex and nationality in the NHS-2006, NHS-2011/2012 and NHS-2007 surveys

**Fig. 2:**
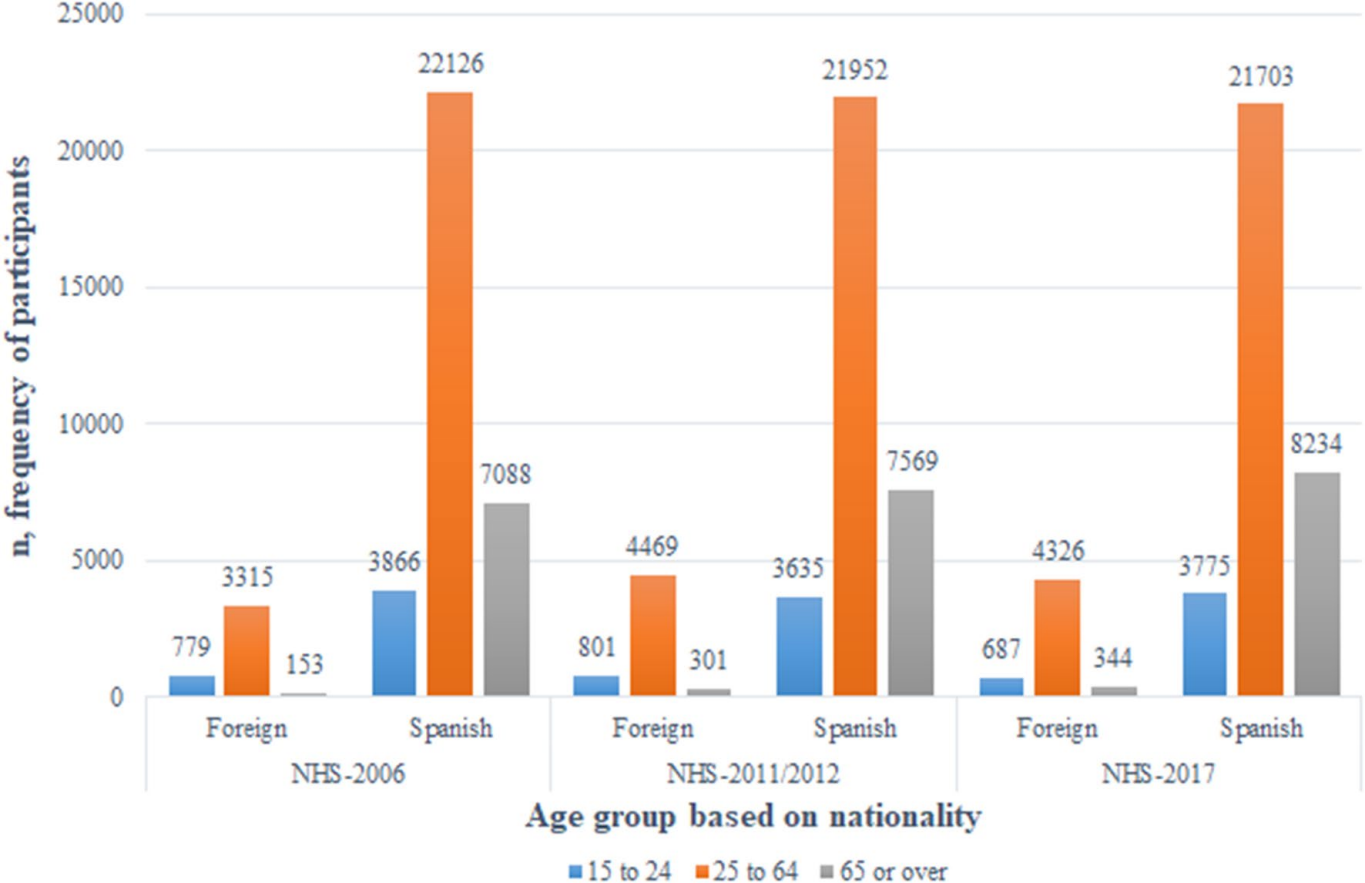
Distributions of the participants according to age group and nationality in the NHS-2006 (the first age group contains participants aged between 16 and 24 years), NHS-2011/2012 and NHS-2007 surveys

All the variables collected had missing values of less than 10%. A total of 115,123 participants from the NHS-2006 (n = 37,327), NHS-2011/12 (n = 38,727) and NHS-2017 (n = 39,069) were analyzed. In NHS-2006 cohort, immigrants represented 11.38% of the sample, with a similar sex ratio (men 5.40% and women 5.98%). In the NHS-2011/12 and NHS-2017, immigrants represented 14.39% and 13.71% of the samples, respectively, with higher proportions of women (7.53% and 7.52%, respectively) than men. In all the surveys, the group aged 25 to 64 years contained the highest proportions of immigrants (Figs. 1, 2).

Table 1 shows the prevalence rates and percentages of the variables self-reported by the participants in the Spanish and immigrant populations in the three surveys: gum bleeding, tooth mobility, tooth extraction, and missing teeth. In the NHS-2006, nationality was significantly associated with all the variables related to periodontal disease (*p* = 0.000 for all). In the NHS-2011/12, nationality was significantly associated with tooth mobility, tooth extraction and missing teeth (*p* = 0.000 for all). Finally, in the NHS-2017, nationality was significantly associated with gum bleeding, tooth mobility and tooth extraction (*p* = 0.000, *p* = 0.001 and *p* = 0.000, respectively).

**Table 1:** Prevalence and proportions of the self-reported variables, namely, bleeding gums, tooth mobility, tooth extraction and missing teeth, in immigrants and Spanish nationals in the NHS-2006, NHS-2011/2012 and NHS-2007

	Bleeding gums					Tooth mobility					Tooth extraction					Missing teeth				
	NHS-2006					NHS-2006					NHS-2006					NHS-2006				
	Immigrant		Spanish		*p* value of the chi-square test	Immigrant		Spanish		*p* value of the chi-square test	Immigrant		Spanish		*p* value of the chi-square test	Immigrant		Spanish		*p* value of the chi-square test
	F	%	F	%		F	%	F	%		F	%	F	%		F	%	F	%	
Yes	994	23.40	6970	21.07	0.000	207	4.87	2562	7.74	0.000	2494	58.72	24,077	72.78	0.000	1886	44.41	15,869	47.97	0.000
No	3130	73.70	25,439	76.90		3890	91.59	29,839	90.20		1633	38.45	8431	25.49		2205	51.92	15,534	46.96	
Not recorded	123	2.90	671	2.03		150	3.53	679	2.05		120	2.83	572	1.73		156	3.67	1677	5.07	
Total	4247	100.00	33,080	100.00		4247	100.00	33,080	100.00		4247	100.00	33,080	100.00		4247	100.00	33,080	100.00	
	NHS-2011/2012					NHS-2011/2012					NHS-2011/2012					NHS-2011/2012				
Yes	923	16.38	5650	17.07	0.076	206	3.70	2179	6.57	0.000	3283	58.93	23,922	72.15	0.000	2538	45.56	16,706	50.39	0.000
No	4623	82.03	27,416	82.85		5340	95.85	30,882	93.14		2263	40.62	9171	27.66		3010	54.03	16,389	49.43	
Not recorded	90	1.60	25	0.08		25	0.45	95	0.29		25	0.45	63	0.19		23	0.41	61	0.18	
Total	5636	100.00	33,091	100.00		5571	100.00	33,156	100.00		5571	100.00	33,156	100.00		5571	100.00	33,156	100.00	
	NHS-2017					NHS-2017					NHS-2017					NHS-2017				
Yes	864	16.13	5585	16.57	0.000	226	4.22	1767	5.24	0.001	3610	67.39	25,031	74.25	0.000	3056	57.05	19,269	57.16	0.518
No	4475	83.54	28,105	83.37		5122	95.61	31,916	94.67		1745	32.57	8665	25.70		2296	42.86	14,426	42.79	
Not recorded	18	0.34	22	0.07		9	0.17	29	0.09		2	0.04	16	0.05		5	0.09	17	0.05	
Total	5357	100.00	33,712	100.00		5357	100.00	33,712	100.00		5357	100.00	33,712	100.00		5357	100.00	33,712	100.00	

The chi-square test was used to assess the associations between nationality and each of the self-reported variables. A *p* value < 0.05 was considered statistically significant. F: frequency; %: percentage

### Self-reported bleeding gums

In the NHS-2006, immigrants had higher odds of bleeding gums than Spanish nationals (OR = 1.65, 95% CI 1.38–1.99, p = 0.000). However, immigrant women (OR = 0.81, 95% CI 0.69–0.94, *p* = 0.006) as well as immigrants aged 25–64 years (OR = 0.61, 95% CI 0.51–0.74, *p* = 0.000) were less likely to have gum bleeding than their counterparts. In the NHS-2017, immigrants aged 25–64 years (OR = 0.77, 95% CI 0.62–0.96, *p* = 0.022) and 65 years and older (OR = 0.49, 95% CI 0.29–0.82, *p* = 0.000) were less likely to have bleeding gums than immigrants in the other age groups (Table 2).

**Table 2:** Multinomial logistic regression of the NHS-2006 (n = 39,069, *p* = 0.000) and NHS-2017 (n = 39,069, *p* = 0.000) for variable gum bleeding

Predictors	NHS-2006			NHS-2017		
	OR	95% CI	*p* value	OR	95% CI	*p* value
*Nationality*						
Spanish	1.00	–	–	1.00	–	–
Immigrant	1.65	1.38–1.99	0.000	1.09	0.87–1.36	0.460
*Age*						
16−24 years	1.00	–	–	1.00	–	–
25–64 years	0.95	0.87–1.02	0.169	1.04	0.95–1.14	0.389
65 or over	0.40	0.36–0.44	0.000	0.46	0.41–0.51	0.000
*Sex*						
Men	1.00	–	–	1.00	–	–
Women	1.23	1.17–1.30	0.000	1.20	1.13–1.27	0.000
*Interaction: sex* * *nationality*						
Man * Spanish	1.00	–	–	1.00	–	–
Woman * immigrant	0.81	0.69–0.94	0.006	0.99	0.85–1.17	0.943
*Interaction: age* * *nationality*						
16–24 years * Spanish	1.00	–	–	1.00	–	–
25–64 years * immigrant	0.61	0.51–0.74	0.000	0.77	0.62–0.96	0.022
65 or over * Immigrant	1.10	0.69–1.74	0.693	0.49	0.29–0.82	0.000

### Self-reported tooth mobility

In the NHS-2017, the interactions between nationality and sex as well as nationality and age were not included in the multinomial logistic regression model (LR = 8.06, *p* = 0.234). The data of the immigrant cohorts in the three surveys were not significant (Table 3).

**Table 3:** Multinomial logistic regression of the NHS-2006 (n = 37,327, *p* = 0.000), NHS-2011/2012 (n = 38,727, *p* = 0.000) and NHS-2017 (n = 39,069, *p* = 0.000) for dental mobility

Predictors	NHS-2006			NHS-2011/2012			NHS-2017		
	OR	95% CI	*p* value	OR	95% CI	*p* value	OR	95% CI	*p* value
*Nationality*									
Spanish	1.00	–	–	1.00	–	–	1.00	–	–
Immigrant	0.97	0.56–1.66	0.899	1.22	0.66–2.24	0.520	0.87	0.75–1.00	0.055
*Age*									
16–24 years	1.00	–	–	1.00	–	–	1.00	–	–
25–64 years	3.27	2.64–4.05	0.000	3.81	2.92–4.96	0.000	3.61	2.81–4.64	0.000
65 or over	5.75	4.62–7.17	0.000	7.71	5.89–10.09	0.000	5.11	3.95–6.62	0.000
*Sex*									
Men	1.00	–	–	1.00	–	–	1.00	–	–
Women	1.05	0.96–1.13	0.280	1.12	1.02–1.22	0.013	1.03	0.94–1.13	0.462
*Interaction: sex* * *nationality*									
Man * Spanish	1.00	–	–	1.00	–	–	NA	NA	NA
Woman * Immigrant	0.97	0.72–1.30	0.822	0.81	0.61–1.09	0.170	NA	NA	NA
*Interaction: age* * *nationality*									
16 to 24 years * Spanish	1.00	–	–	1.00	–	–	NA	NA	NA
25–64 years * Immigrant	0.73	0.42–1.27	0.270	0.57	0.31–1.05	0.072	NA	NA	NA
65 or over * Immigrant	1.18	0.58–2.40	0.644	0.70	0.34–1.44	0.332	NA	NA	NA

### Self-reported tooth extraction

In the NHS-2006, immigrants had higher odds of tooth exodontia than Spanish nationals (OR = 1.50, 95% CI 1.26–1.79, *p* = 0.000). However, immigrant women (OR = 0.69, 95% CI 0.60–0.80, *p* = 0.000) and immigrants aged 25–64 years (OR = 0.45, 95% CI 0.38–0.54, *p* = 0.000) and 65 years and older (OR = 0.32, 95% CI 0.21–0.48, *p* = 0.000) were less likely to require tooth exodontia than their counterparts. In the NHS-2011/12, immigrants aged 25–64 years (OR = 0.64, 95% CI 0.54–0.77, *p* = 0.000) and 65 years and older (OR = 0.66, 95% CI 0.47–0.93, *p* = 0.017) showed a lower probability of tooth exodontia than immigrants in the other age groups. In the NHS-2017, immigrants aged 25–64 (OR = 0.69, 95% CI 0.57–0.83, *p* = 0.000) and 65 years and older (OR = 0.49, 95% CI 0.35–0.70, *p* = 0.000) showed a lower probability of tooth exodontia than immigrants in the other age groups (Table 4).

**Table 4:** Multinomial logistic regression of the NHS-2006 (n = 37,327, *p* = 0.000), NHS-2011/2012 (n = 38,727, *p* = 0.000) and NHS-2017 (n = 39,069, *p* = 0.000) for dental tooth extraction

Predictors	NHS-2006			NHS-2011/2012			NHS-2017		
	OR	95% CI	*p* value	OR	95% CI	*p* value	OR	95% CI	*p* value
*Nationality*									
Spanish	1.00	–	–	1.00	–	–	1.00	–	–
Immigrant	1.50	1.26–1.79	0.000	0.94	0.78–1.12	0.473	1.14	0.95–1.37	0.169
*Age*									
16–24 years	1.00	–	–	1.00	–	–	1.00	–	–
25–64 years	6.35	5.89–6.84	0.000	6.20	5.75–6.69	0.000	7.12	6.60–7.68	0.000
65 or over	16.42	14.83–18.19	0.000	14.70	13.33–16.20	0.000	23.25	20.98–25.76	0.000
*Sex*									
Men	1.00	–	–	1.00	–	–	1.00	–	–
Women	1.38	1.31–1.46	0.000	1.09	1.04–1.15	0.001	1.06	1.00–1.12	0.038
*Interaction: sex* * *nationality*									
Man * Spanish	1.00	–	–	1.00	–	–	1.00	–	–
Woman * Immigrant	0.69	0.60–0.80	0.000	0.99	0.87–1.12	0.834	1.04	0.91–1.19	0.529
*Interaction: age* * *nationality*									
16–24 years * Spanish	1.00	–	–	1.00	–	–	1.00	–	–
25–64 years * Immigrant	0.45	0.38–0.54	0.000	0.64	0.54–0.77	0.000	0.69	0.57–0.83	0.000
65 or over * Immigrant	0.32	0.21–0.48	0.000	0.66	0.47–0.93	0.017	0.49	0.35–0.70	0.000

In the NHS-2006, immigrants had higher odds of missing teeth (OR = 1.44, 95% CI 1.19–1.75, *p* = 0.000). However, immigrant women (OR = 0.66, 95% CI 0.58–0.75, *p* = 0.000) as well as immigrants between 25 and 64 years of age (OR = 0.73, 95% CI 0.60–0.89, *p* = 0.002) and 65 or older (OR = 0.61, 95% CI 0.42–0.90, *p* = 0.012) were less likely to have missing teeth than their counterparts. In the NHS-2011/12, immigrants had higher odds of missing teeth than Spanish nationals (OR = 1.30, 95% CI 1.07–1.59, *p* = 0.008). However, immigrants 25 to 64 years of age (OR = 0.60, 95% CI 0.49–0.73, *p* = 0.000) and 65 years and older (OR = 0.67, 95% CI 0.49–0.90, *p* = 0.009) were less likely to have missing teeth than immigrants in the other age groups (Table 5).

**Table 5:** Multinomial logistic regression of the NHS-2006 (n = 37,327, *p* = 0.000) and NHS-2011/2012 (n = 38,727, *p* = 0.000) for missing teeth

Predictors	NHS-2006			NHS-2011/2012		
	OR	95% CI	*p* value	OR	95% CI	*p* value
*Nationality*						
Spanish	1.00	–	–	1.00	–	–
Immigrant	1.44	1.19–1.75	0.000	1.30	1.07–1.59	0.008
*Age*						
16–24 years	1.00	–	–	1.00	–	–
25–64 years	3.99	3.64–4.38	0.000	5.57	5.09–6.09	0.000
65 or over	2.94	2.66–3.25	0.000	5.87	5.32–6.46	0.000
*Sex*						
Men	1.00	–	–	1.00	–	–
Women	1.22	1.17–1.28	0.000	0.93	0.89–0.97	0.002
*Interaction: sex* * *nationality*						
Man * Spanish	1.00	–	–	1.00	–	–
Woman * immigrant	0.66	0.58–0.75	0.000	1.10	0.98–1.24	0.110
*Interaction: age* * *nationality*						
16–24 years * Spanish	1.00	–	–	1.00	–	–
25–64 years * immigrant	0.73	0.60–0.89	0.002	0.60	0.49–0.73	0.000
65 or over * immigrant	0.61	0.42–0.90	0.012	0.67	0.49–0.90	0.009

## Discussion

The main findings of this study showed that among the self-reported variables of periodontal disease, there was a similar proportion of gum bleeding between immigrants (between 16 and 23%) and Spanish nationals (between 17 and 21%). The same result was observed for dental mobility, in which immigrants (between 4 and 5%) and Spanish nationals (between 5 and 8%) presented similar proportions. The multivariate model showed that in the 2006 survey, immigrants reported bleeding 1.65 times more frequently than Spanish participants. However, the interactions between immigrant status and female sex as well as immigrant status and age 25–64 years behaved as protective factors against bleeding gums. On the other hand, in the 2017 survey, immigrants did not have increased odds of bleeding gums, and in this case, the interaction between immigrant status and age 65 years or older acted as a protective factor.

For dental mobility, the regression models did not show significant associations with the sociodemographic predictor variables such as age and sex and their interactions with nationality.

There was a high proportion of immigrants aged between 25 and 65 years; this result coincides with those of other studies in which the migration of people of working age to other countries is frequent [23, 24].

A strength of our study is the good representativeness of the sample in Spain due to the large sample size included in the three surveys. In addition, sociodemographic variables, age and sex were controlled for, as they had possible confounding effects on nationality and variables related to the presence or condition of periodontal disease.

Another strength was the evaluation among the national population and immigrants in different age groups and different surveys, which shows a certain trend in the evolution of the periodontal disease in immigrants.

There are several limitations in our study. First, this study employed a cross-sectional design; therefore, causal relationships between the variables could not be established. Consequently, the results should be interpretated with caution [25].

Second, the countries of origin of all the participants included in the immigrant cohort were not considered. This can introduce bias, as different cultural factors can influence the outcomes in this group. Other surveys carried out in populations in Spain report an increased probability of gum bleeding in Moroccan women (OR = 3.61; 95% CI 1.83–7.15) [26]. Since this study included a global sample of immigrants, cultural subgroup analyses could not be performed.

Third, the data sources were self-reported surveys. Other studies recently carried out in Spain where periodontitis is self-reported in a much smaller sample size (231 participants [8] and 112 participants [7]) were possible to validate the results with a full-mouth periodontal examination. On the other hand, some studies suggest that the information obtained from self-reported questionnaires may be strongly influenced by educational level and other socioeconomic characteristics [27]. In our case, due to the large sample size, the results of the respondents were not validated in a clinical examination. Nor were validated surveys used, and the biases of the generalized participation of any person should be considered. It must be taken into account that the objective of the surveys in our study arises within the health policies of our country to have indicators to achieve adequate planning and adoption of public health measures. Therefore, the participants' questions arise from the need to collect information that any person can answer about the state of their teeth and molars. The generalization of National Health Surveys has already been used in other studies published in Spain where the association between periodontal disease and chronic obstructive pulmonary disease was evaluated [25].

Finally, in relation to the variables studied, only some sociodemographic factors, such as sex and age, were controlled for, but there may be others that influence oral health, such as smoking [28, 29], psychological stress [29], diabetes, pregnancy [30], menopause, rheumatoid arthritis, systemic lupus erythematosus [31], vitamin C consumption [32, 33] or other nutritional factors [33, 34]. Other self-reported surveys to evaluate periodontitis collect more factors that can influence periodontal diseases, such as sociodemographic characteristics, oral hygiene behaviors, and periodontal measurements [9, 10]. In relation to the variables selected to describe the presence of periodontal disease, the absence of other measurements, such as probing depth or the presence of bone loss [35], which would aid in a more precise diagnosis, were not provided, so there may be a bias related to diagnosis.

The data associated with the variables tooth exodontia and missing teeth collected should be interpreted with caution since they may represent an indirect cause of periodontal disease, and periodontal disease may be due to other reasons. The percentage of tooth loss was always increased in the groups aged 25 and 65 years, which may be due to a diet rich in carbohydrates and/or the presence of periodontal disease [36, 37].

## Conclusion

Immigrants in Spain have a lower probability of developing signs associated with periodontal disease than the Spanish population. In the immigrant cohort, where this pathology is observed less frequently, female sex and adult and elderly age ranges play essential roles.

## Data Availability

The datasets analysed during the current study are available in the repository of the National Institute of Statistics of Spain, https://www.ine.es/dyngs/INEbase/es/operacion.htm?c=Estadistica_C&cid=1254736176783&menu=metodologia&idp=1254735573175. Also, the dataset analysed during the current study are available from the corresponding author on reasonable request.
